# A comprehensive global genotype–phenotype database for rare diseases

**DOI:** 10.1002/mgg3.262

**Published:** 2016-11-23

**Authors:** Daniel Trujillano, Gabriela‐Elena Oprea, Yvonne Schmitz, Aida M. Bertoli‐Avella, Rami Abou Jamra, Arndt Rolfs

**Affiliations:** ^1^ Centogene AGRostockGermany; ^2^ Institute of Human GeneticsUniversity of Leipzig Hospitals and ClinicsLeipzigGermany; ^3^ Albrecht‐Kossel‐Institute for NeuroregenerationMedical University RostockRostockGermany

**Keywords:** Clinical diagnostics, HPO, rare disease, variant database

## Abstract

**Background:**

The ability to discover genetic variants in a patient runs far ahead of the ability to interpret them. Databases with accurate descriptions of the causal relationship between the variants and the phenotype are valuable since these are critical tools in clinical genetic diagnostics. Here, we introduce a comprehensive and global genotype–phenotype database focusing on rare diseases.

**Methods:**

This database (CentoMD ^®^) is a browser‐based tool that enables access to a comprehensive, independently curated system utilizing stringent high‐quality criteria and a quickly growing repository of genetic and human phenotype ontology (HPO)‐based clinical information. Its main goals are to aid the evaluation of genetic variants, to enhance the validity of the genetic analytical workflow, to increase the quality of genetic diagnoses, and to improve evaluation of treatment options for patients with hereditary diseases. The database software correlates clinical information from consented patients and probands of different geographical backgrounds with a large dataset of genetic variants and, when available, biomarker information. An automated follow‐up tool is incorporated that informs all users whenever a variant classification has changed. These unique features fully embedded in a CLIA/CAP‐accredited quality management system allow appropriate data quality and enhanced patient safety.

**Results:**

More than 100,000 genetically screened individuals are documented in the database, resulting in more than 470 million variant detections. Approximately, 57% of the clinically relevant and uncertain variants in the database are novel. Notably, 3% of the genetic variants identified and previously reported in the literature as being associated with a particular rare disease were reclassified, based on internal evidence, as clinically irrelevant.

**Conclusions:**

The database offers a comprehensive summary of the clinical validity and causality of detected gene variants with their associated phenotypes, and is a valuable tool for identifying new disease genes through the correlation of novel genetic variants with specific, well‐defined phenotypes.

## Introduction

The extraordinary progress of massive parallel sequencing technologies has produced one of the major scientific breakthroughs in recent years. Next‐Generation Sequencing (NGS) technologies represent an important milestone in genomics and in clinical genetics, revolutionizing the way geneticists screen for disease‐causing genetic variants. NGS in combination with robust bioinformatics analyses can deliver fast and detailed genetic information, providing a rapid, cost‐effective approach for identifying genetic variants and enabling more rapid genetic diagnosis (Hennekam and Biesecker 2012). However, the current ability to discover a genetic variation in a patient genome is running far ahead of the ability to interpret that variation. The numbers of genotype calls generated by NGS are in the order of 10^4^ per exome, 10^5^ for the combined exomes of a small family, and 10^6^ per genome (Adams et al. 2012). Detected variants must therefore undergo a filtering cascade to identify those that are clinically relevant. Therefore, the success of NGS in medical genetics hinges on the accuracy of methods used to distinguish causal from benign alleles. In this regard, databases with accurate genotype–phenotype information become a critical tool in the interpretation of NGS data (Johnston and Biesecker 2013).

Obtaining accurate genetic diagnoses is still a highly demanding task because only a small proportion of known genetic variants have been described or precisely annotated (Abecasis et al. 2012). Furthermore, new genetic variants are revealed in each new patient investigated. In the context of genetically determined disorders, these variants are very rare and are typically annotated as “variant of unknown significance” (VUS; Rehm et al. 2015). In such cases, diagnoses are left uncertain and proper treatment options remain hard to evaluate. Medical professionals therefore need to obtain all available knowledge about a patient's gene variants in order to establish the most accurate possible diagnosis.

The development of human gene variant databases began with the collection of globin gene mutations (Baglioni 1962; Hardison et al. 1998). Ever since, human gene variant databases for rare Mendelian disorders have become crucial in today's data‐driven genetic diagnostics, and play a role in life‐and‐death decisions (George et al. 2008). Such databases consist of aggregated genotype–phenotype data and assist enquiring laboratories or clinicians, making sequence variant interpretation easier and more cost‐effective (Vail et al. 2015).

Here, we introduce a comprehensive and global genotype–phenotype database (Centogene's Mutation Database, CentoMD^®^) that focuses primarily on human rare diseases. This resource is a holistic database that combines human phenotype ontology (HPO)‐based and carefully curated genotype information gathered from detailed medical reports of patients worldwide, processed for genetic diagnostic workflows at Centogene AG (Rostock, Germany). This means that every variant reported in CentoMD^®^ is linked to at least one clinically described and consented case analyzed through a standardized workflow with accredited quality. The database incorporates browser‐based software that enables access to its comprehensive and curated repository. It correlates clinical information of consented patients and probands of different geographical backgrounds with a large dataset of genetic variants plus, when available, biomarker information.

CentoMD^®^ is a rapidly growing pay‐per‐use database with newly generated data being imported in a quarterly basis. The main goals of developing this database are to aid trained professionals evaluate genetic variants, to enhance the validity of genetic analytical workflows, and to assist in the genetic diagnosis, counseling, and evaluation of treatment options for patients with hereditary diseases.

## Methods

### Data acquisition and curation

The curation of genetic data involves the collection, association, update and review of genetic and phenotypic data of genetically analyzed patients in a standardized, structured format into a curation repository. This system utilizes a combination of computer‐based tools and manual review to maximize accuracy, efficiency, and data quality.

Database curators are biologists and physicians with strong background in human genetics. They continuously undergo extensive training to ensure curation consistency and standardization. Systematic, repeated control of clinical and genetic content is performed to ensure that items are properly associated and interpreted, and that there are no inconsistencies and/or discrepancies compared with in‐house observations or external sources. The curation process is finalized by manual approval to confirm that reviewed and curated data agree with in‐house criteria.

Data gathering and curation are conducted using web‐based software that is compliant with Human Genome Nomenclature Committee (HGNC), Human Genome Variation Society (HGVS), and HPO nomenclatures. This allows collection of variants detected in nuclear coding, nuclear noncoding and mitochondrial genes. The software integrates in‐house sample management and analysis systems, and cross‐references external databases to give curators a comprehensive yet straightforward overview of evidence regarding genotype–phenotype correlations.

Data are gathered by a combination of manual submission and data import according to a case‐oriented model, where characteristics belonging to a particular individual (patient information, clinical data, methodology, and detected genetic variants) are stored and associated together. To provide high‐quality data, the curation process is divided into three phases comprising variant‐wise, case‐wise, and warnings‐wise procedures.

#### Curation by variant

To begin the curation process, the variant‐linked information is reviewed to check nomenclature, terminology, accuracy, consistency, and record completeness.

#### Curation by case

In order to start curation by case, all detected variants must be approved to ensure that entries belonging to an individual follow the rules for clinical statement, and that all associated data are in agreement with agreed guidelines. The following factors are considered critical for the clinical statement: variant significance, patient genotype (number of clinically relevant changes, variant zygosity, and location [i.e., cis vs. trans]), inheritance pattern of the disorder, patient gender (for X‐linked diseases), phenotype description, and biomarker levels (if available).

#### Curation by warning

The database generates warnings at different levels (variant, case, gene, database levels) to detect errors, invalid terms and nomenclatures, and inconsistencies, and can provide hints where updates and reviews are necessary. Mostly these warnings are due to additional evidence in the form of medical reports, additional published articles, or other databases. Each warning is manually resolved and documented in detail. Whenever additional evidence becomes available, variants are revised and reclassified accordingly. At least quarterly, all approved cases are anonymized and released to the database.

### Ethical compliance

All patients provided informed consent before inclusion in the CentoMD^®^ database. Once included, all patient data are fully anonymized.

### Classification of genetic variants

The classification of genetic germline variants is done according to ACMG guidelines (Richards et al. 2015). Detected genetic variants are first classified into one of three classes concerning their likelihood to predispose to, or to cause, the observed phenotype/disease: clinically relevant variants (CRV); clinically irrelevant variants (CIV); and VUS.

The CRV class includes the following subclasses: pathogenic, likely pathogenic, risk factor, and modifier. Classification is based on variants' impact on disease presence, severity or increased susceptibility. Variants that are subclassified as pathogenic or likely pathogenic according to the Human Gene Mutation Database (HGMD^®^) are reassessed by evaluation of the original published papers, and the variant is reclassified accordingly in consideration of existing in‐house data. Novel loss‐of‐function (LoF) and missense variants that lead to novel amino acid changes where a previous pathogenic variant was described (highly conserved and predicted as damaging by in silico tools) are subclassified as likely pathogenic.

To evaluate missense variants, PolyPhen‐2, SIFT, MutationTaster, and Align GVGD are used to apply a combination of evolutionary conservation and protein structure/function evaluation algorithms. At least three tools need to be evaluated. For WES cases, all automatically included tools are considered. To evaluate splicing, SSF, Human Splice finder (HSF), and MaxEntScan are applied. A splicing report window allows entry of data from any significant changes in the splicing score predicted by any program: threshold differences are >5% for SSF, >15% for MaxEnt or >10% for HSF. It is important to evaluate if the predicted skipped exon is physiologically alternatively spliced out, if it occurs in frame, and if there are important domains in this region. In particular regions including upto acceptor 12 bp intron, 2 bp exon or donor 3 bp exon, 8 bp intron should be evaluated. Thresholds for the recognition of a natural splice site by the splicing programs are: MES > 3, SSF > 60, HSF > 80. The following tools are applied in cases featuring nucleotide conservation: GERP (upto 6.16, considering >2 as conserved and >4 as highly conserved); PhyloP (considering >2.5 as highly conserved); and PhastCons (considering 0–1, >0.9 as highly conserved). At least two of these findings need to be considered.

*De novo* variants that lead to insertions or deletions within a nonrepetitive region, or that lead to a *de novo* amino acid change (if highly conserved and predicted as damaging by in silico tools) are also considered likely pathogenic, along with variants that, according to our data, are found in at least three unrelated, similarly affected patients.

The CIV class includes the following subclasses: benign, likely benign, and disease‐associated polymorphisms, and Centogene‐published (likely) benign‐published as (likely) pathogenic (i.e., likely benign according to CentoMD^®^). Variants are considered as CIV, based on their high frequency in population(s) as opposed to lack of observed impact on disease presence/severity/susceptibility or nonsegregation and/or co‐occurrence, etc. Disease‐associated polymorphisms are included for disorders with known multigene, complex inheritance. Such variants must have a maximum minor allele frequency (MAF) of 5% in public databases and the association should be replicated by at least two independent studies or in one study with functional evidence. When the internal evidence regarding the clinical significance of a variant is inconsistent compared with external resources, the subclass “Centogene‐published (likely) benign‐published as (likely) pathogenic” is used to highlight the importance of this observation. Variants in this subclass were detected in at least two unrelated, healthy/unaffected individuals, taking into account age at disease onset, for example.

The VUS class includes rare variants, irrespective of reports in the literature, with unknown risk of developing or causing disease, in cases where prediction software shows inconsistent effects, or when family studies do not support the variant's impact on phenotype.

Variant reevaluation and reclassification is a key feature of the database and is performed at least quarterly to assure that only the most updated and clinically useful information are included. When enough internal or external evidence becomes available, the reclassification process is initiated and the potential pathogenicity of variants is reevaluated: customers are informed accordingly.

In order to assure the high data quality in CentoMD^®^ database, the detected variants are divided into three quality classes: (1) classified and curated (++) for variants assigned to a clinical significance class based on the genotype–phenotype correlation and manually curated following strictly the ACMG guidelines and internal expertise; (2) classified (+) for variants assigned to a clinical significance class according to ACMG guidelines but not yet manually curated due to not enough evidence that genotype–phenotype correlation exists; (3) unclassified (0) for variants not yet assigned to any clinical significance class.

### Whole‐exome dataset and phenotype‐based queries

The CentoMD^®^ v3.1 phenotype to genotype module provides access to the aggregated WES datasets and linked HPO‐based clinical information of >8000 consented cases diagnosed at Centogene AG. The genotype–phenotype module includes CRV and VUS variants identified to be associated or probably associated with the clinical symptomatology observed in each individual case.

This module allows searching for individuals sharing a given set of signs and symptoms based on HPO. The user starts defining a set of HPO terms of interest and the system will calculate similarity scores across all cases in the database. Known database cases are presented based on similarity scores (and associated *P*‐values; Deng et al. 2015). Detailed HPO descriptions are also provided for each case, allowing users to decide which database cases are of interest, and search for candidate genes and their associated variants. After the selection of samples, the process of variant selection follows the same rationality as that explained above for the WES database, with the only difference being that the variant search is limited to a list of user‐defined cases.

## Results

### Database statistics

The CentoMD^®^ 3.1 database covers more than 2500 disease genes, including nuclear coding, nuclear noncoding, and mitochondrial genes screened by Sanger, NGS, fragment length analysis (FLA), multiplex ligation‐dependent probe amplification (MLPA), and/or quantitative polymerase chain reaction analysis (qPCR). Each gene available in the database is linked to the corresponding disease(s) and/or condition(s) according to the Online Mendelian Inheritance in Man (OMIM) catalog or on the basis of medical reports and the corresponding mode of inheritance.

The patient cohort of the database is highly heterogeneous and originates from more than 100 countries. Current cohort characteristics are summarized in Table 1. To date, more than 100,000 genetically screened individuals (including probands and family members) are documented in CentoMD^®^ version 3.1. For almost 16,200 patients (17.5%), the clinical suspicion of a rare disease has been genetically confirmed (“cases”) using the database, and over 8800 individuals (9.5%) have been identified as a carrier of at least one clinically relevant variant (“carriers”).

**Table 1:** CentoMD^®^ cohort characteristics

Characteristics	%
Individuals from testing and systematic screening programs (Rolfs et al. 2013)	
Cases (genetically confirmed diagnosis)	17.5
Carriers (of a clinically relevant genetic variant)	9.9
No confirmation of a hereditary disorder	72.6
Gender	
Females	51.8
Males	48.2
Geographic distribution	
Europe	58.3
Middle East	18.8
South and Central America	15.2
North America	5.2
Other regions	4.0
Age at genetic diagnosis [years] (cases and carriers)	
Prenatal	0.4
<3	16.7
3–10	15.4
11–18	9.4
19–44	39.3
45–64	14.9
>65	3.9

**Figure 1:**
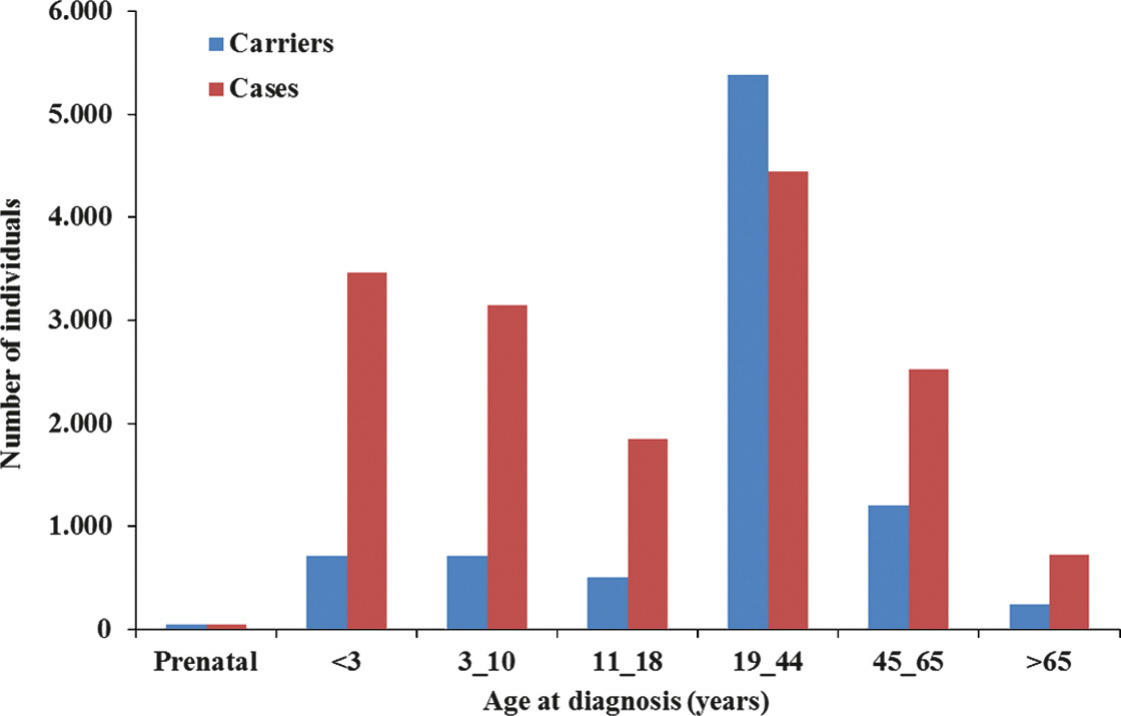
Distribution of confirmed cases and carriers by age at genetic diagnosis.

**Figure 2:**
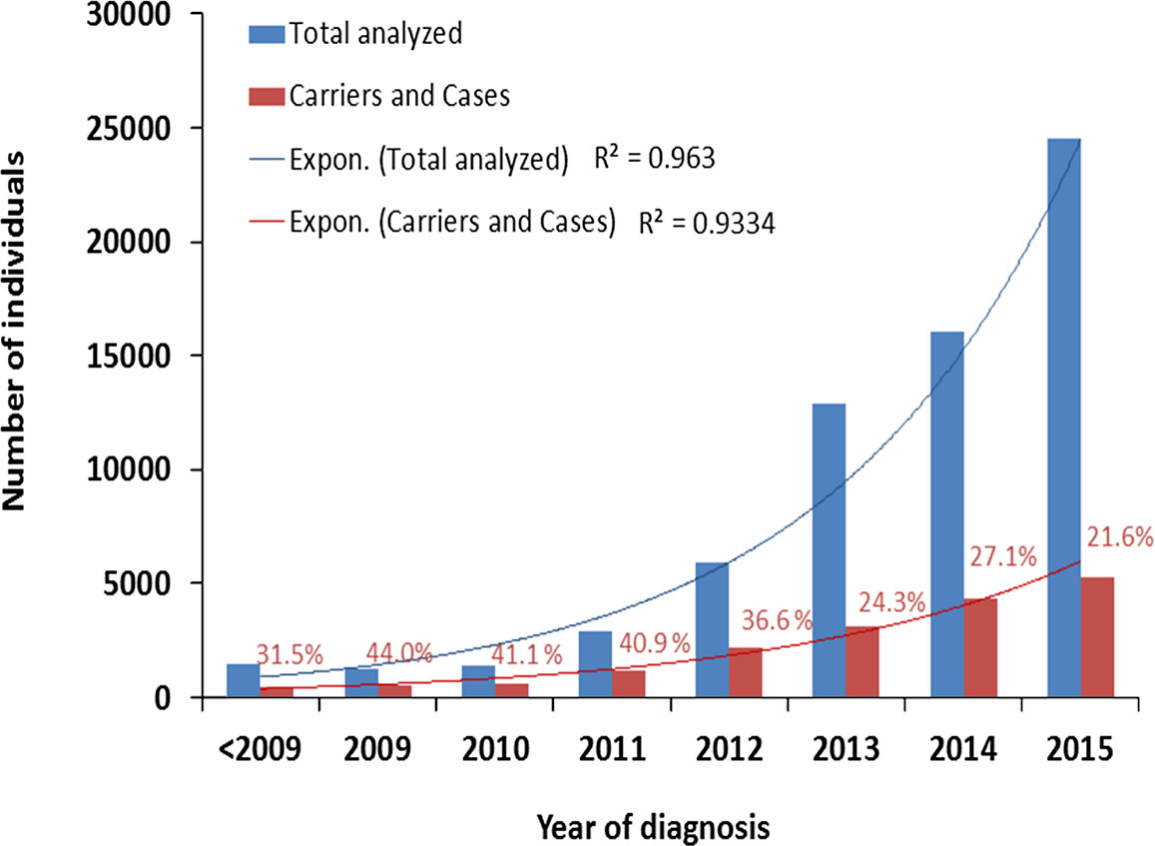
Annual evolution of screened and positive cases in CentoMD ^®^.

The average age at genetic diagnosis for cases affected by an autosomal‐dominant inherited disease is 29.4 ± 15.3 years; for cases affected by a recessive inherited disease, it is 12.3 ± 21.6 years. For the x‐linked diseases, the observed age at diagnosis is 25.3 ± 21.6 years for male patients and 38.0 ± 20.8 years for clinically affected female patients. In total, the observed age at genetic diagnosis ranges from the prenatal stage to >80.0 years (Fig. 1). In the period between 2007 and 2015, the total annual screening rate increased by an average of 53.0% (Fig. 2).

### Genetic variants

The CentoMD^®^ database provides information about the genotype–phenotype correlation based on clinical cases that are genetically screened. As of September 2016 (version 3.1), the database contains more than 42,700 unique, manually curated variants (++) detected in more than 100,000 consented patients. All variant‐type annotations provide mapping to genomic coordinates (genome build hg19) and strictly adhere to HGVS guidelines for both small and gross gene rearrangements (den Dunnen and Antonarakis 2000). Variant annotations originate from medical reports issued at Centogene AG, and are curated through a highly standardized process (Table 2). Additionally, more than 850,000 WES variants are included under the classified only variants (+).

**Table 2:** Types of genetic variants in CentoMD^®^ database

Characteristics	%
Annotation classes^a^	
CRV	20.4
VUS	11.9
CIV	67.7
Variant types: CRV and VUS	
Missense	57.6
Frameshift	12.6
Nonsense	11.0
Splicing	10.2
In‐frame	3.1
Gross/gene deletions and rearrangements	2.8
Repeat/expansion	1.7
Other	1.0
Association of CRV and VUS with clinical evidence	
Detailed clinical description following the HPO	52.7
General clinical description	22.9
Biochemical evidence	3.5
No association(s)	20.9
Published status: CRV and VUS	
Published in CentoMD^®^ only	56.9
Published in the literature (PMID available)	43.1

a Only classified and manually curated variants (++) included.

CRV, clinically relevant variants; CIV, clinically irrelevant variants; VUS, variant of unknown significance.

**Figure 3:**
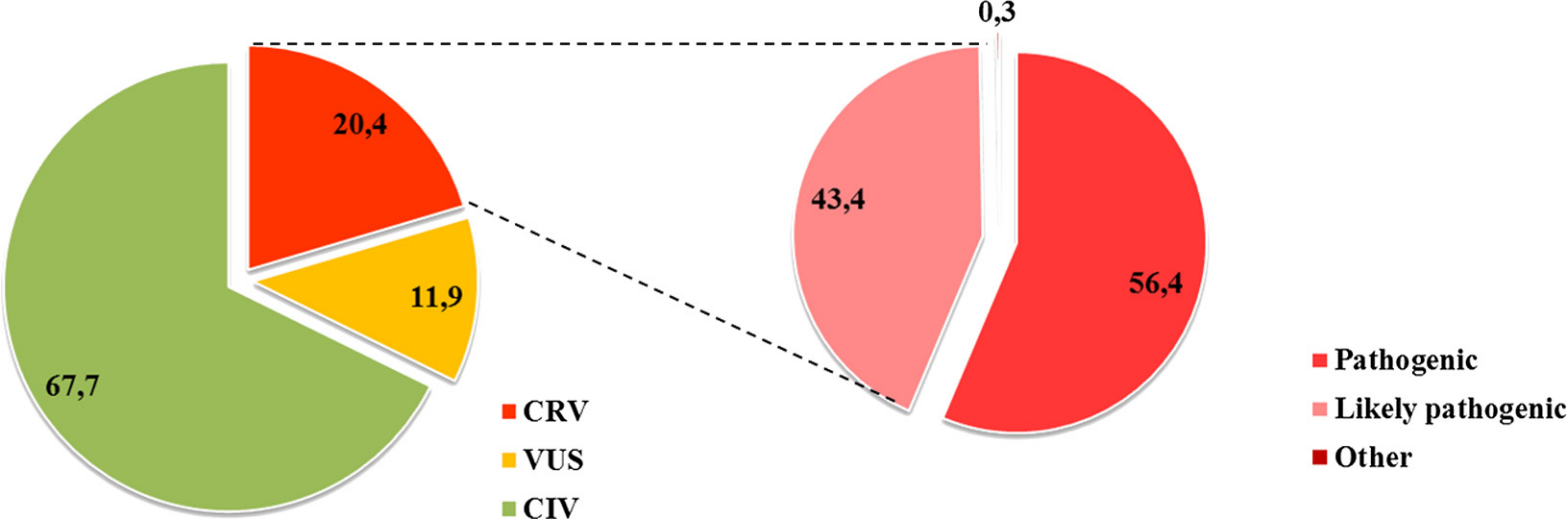
Classification of genetic variants according to their clinical significance in CentoMD ^®^. “Other” CRV variants include risk factors and modifiers. CRV, clinically relevant variant, CIV, clinically irrelevant variant, VUS, variant of unknown significance.

Among all curated ++ variants, 67.7% are classified as CIV (i.e., with no impact on patient clinical phenotype) and 20.4% are classified as CRV (i.e., with a clear impact on clinical symptomatology or disease severity). The remaining 11.9% are variants where additional evidence is required in order to classify them either into relevant or irrelevant variants (i.e., VUS; Fig. 3, left panel). Among the CRV, 56.4% are pathogenic variants, whereas 43.4% are likely pathogenic variants. The remaining 0.3% of variants comprises of risk factors and modifiers (Fig. 3, right panel).

A total of 2.7% of ++ variants that have been published elsewhere as pathogenic variants (i.e., associated with a PMID) have been reclassified as neutral variants at Centogene AG based on the internally available clinical evidence.

The following case illustrates one of such variant reclassifications – a patient suspected to have glycine encephalopathy in whom two variants in the *GLDC* (OMIM 238300) gene were detected; NM_000170.2; c.2113G>A (p.Val705Met) and c.2182G>A (p.Gly728Arg). The variant c.2113G>A (p.Val705Met) was previously reported as likely pathogenic in independent literature (Conter et al. 2006), while c.2182G>A has not been reported previously. However, it is affecting a highly conserved nucleotide and amino acid and all in silico programs‐predicted pathogenicity. Also, at the same position, another substitution has been reported as pathogenic (Kure et al. 2006). This suggested that the diagnosis could be genetically confirmed by two likely pathogenic variants. However, parental carrier testing revealed that both variants were present in the healthy father (in cis). Consequently, insertion deletion analysis was performed, and indeed a heterozygous deletion of exons 1–15 of the *GLDC* was identified in the mother and the affected child. Reevaluation of the missense variants, based on this information and the in the meanwhile available minor allele frequencies (MAF) in public databases led to the conclusion that the variant c.2113G>A (p.Val705Met) is probably not pathogenic (high MAF of 0.027 in Puerto Ricans, 1000 Genomes (Abecasis et al. 2012)). Thus, c.2113G>A (p.Val705Met) was reclassified from pathogenic to likely neutral.

The nature of ++ genetic variants classified as either CRV or VUS ranges from missense (57.6%) to frameshift (12.6%) nonsense (11.0%), splicing (10.2%), or in‐frame variants (3.1%), gene/gross deletions/rearrangements (2.8%), and repeats/extensions (1.7%). The “other” types (1.0%) include deletions, conversions, start loss, stop loss changes etc. (Table 2).

**Figure 4:**
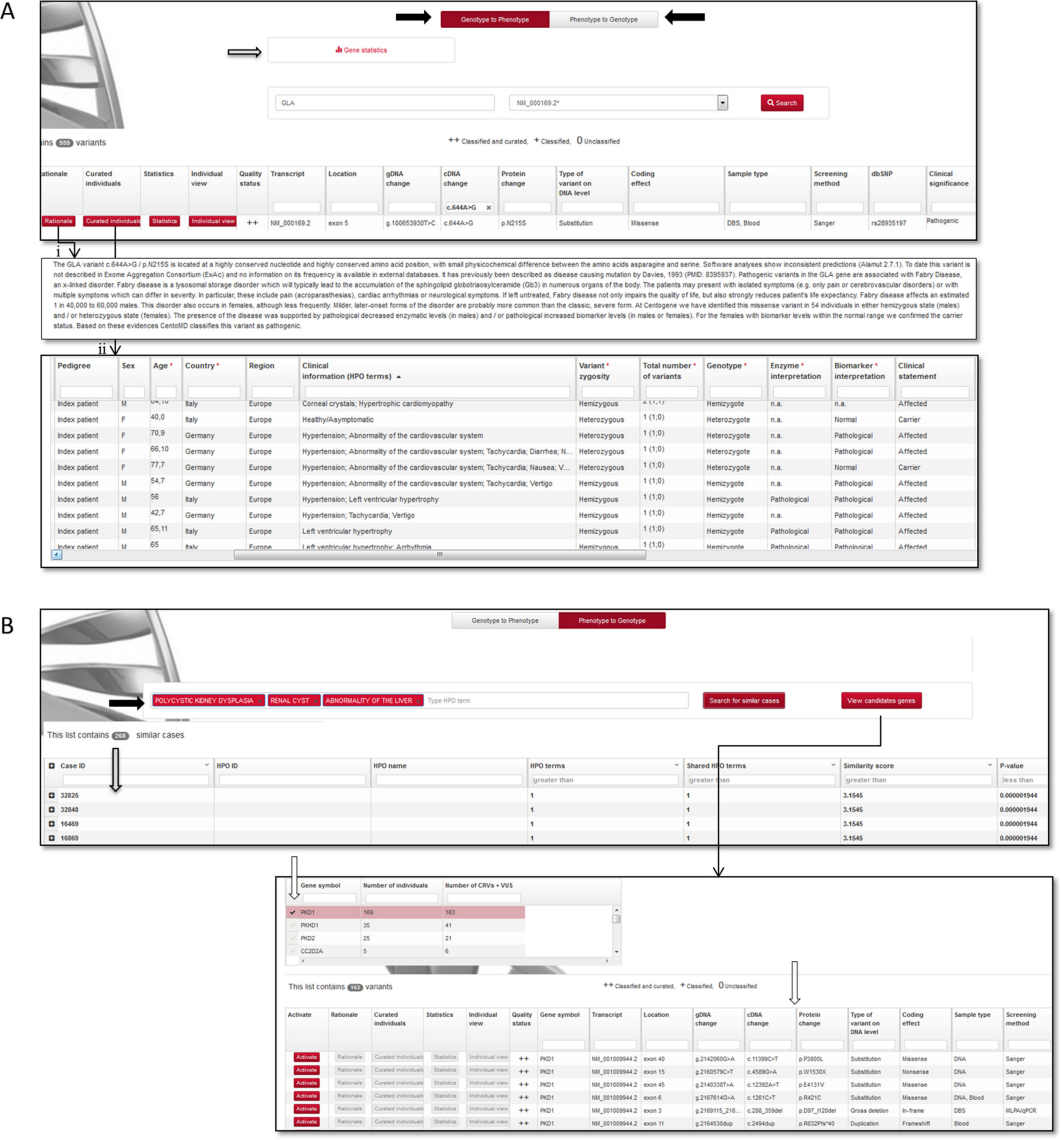
Viewing results in CentoMD database. A) GLA c.644A>G variant as displayed by the Genotype‐Phenotype module. Black arrows indicate the two search modules: Genotype to Phenotype and Phenotype to Genotype. The result table contains variant detailed information and four additional options: rationale (1), curated individuals; (2), statistics and individual view. Gray arrow: gene‐based statistics. B) Phenotype search using HPO terms: polycystic kidney disease, renal cysts, and abnormality of the liver, as displayed by Phenotype‐Genotype module. Black arrows indicate the search field using HPO terms. Gray arrow: table with similar cases. White arrow: candidate genes most likely explaining the clinical symptoms used to initiate the search. Arrow: result table of variants (CRV and VUS) identified in similar cases sharing the HPO terms used to initiate the search. By variant activation, rationale, curated individuals, statistics, and individual view are available for user.

Genetic variants included in the database are associated with phenotypic descriptions provided by the corresponding physician following HPO nomenclature (Köhler et al. 2014). To date, 52.7% of the CRV and VUS are associated with detailed clinical features described with HPO terms. For 22.9% of the CRV and VUS, the presence of a specific disease was suspected by the corresponding physician, whereas 3.5% of variants are not yet associated with clinical information or the presence of a disease, but are instead linked to additional evidence such as enzymatic activity and/or biomarker levels. More than 6000 CRV and VUS (45%) ++ variants are linked to rationale, a summary comprising the supporting evidence for the corresponding clinical significance according to the ACMG guidelines and internal evidence (Fig. 4).

From almost 13,700 clinically relevant and uncertain variants, 43.1% have previously been described in the literature, whereas 56.9% of the genetic variants are novel variants that have not yet been described or reported as being associated with, or causing any clinical phenotype. Among more than 8600 CRV, 60.7% are already published in the literature, whereas 39.3% are novel disease‐causing variants that are described for the first time in the database. As would be expected, the majority of more than 4400 of the VUS are reported only in the database. In particular, this last group is reviewed and reclassified regularly based on new evidence (new cases, new family members and segregation, co‐occurrence with disease‐causing variants in the same gene or other phenotype‐linked genes, etc.). Altogether, 58.6% of the CRV and VUS in the database have been identified only in a single patient, 18.8% of them in two patients, and 4.7% in more than five patients.

## Discussion

The archiving and curation of genotype–phenotype data is a complex, laborious, and expensive task (Cotton et al. 2007). Data accuracy and currency in relevant databases must be ensured by database curators. Genotype–phenotype databases have tended to present reduced information in efforts to anonymize information for data sharing, or because it is simply not available. However, such simplified information may be misleading (Vail et al. 2015). Moreover, the exponential growth of genetic data following the advent of NGS technologies brings new challenges for database design and curation (George et al. 2008).

For the routine clinical use of gene variant databases, several important requirements need to be fulfilled: (1) a stringent quality management system must be in place to create, process, and interpret the data; (2) a patient population representative for different geographical backgrounds must be covered; (3) in‐depth, standardized annotation of the patient phenotypes and family history should be applied for proper and transparent usage of data in the diagnostic process. However, it is generally well accepted that publicly accessible variant databases present substantial disparities in terms of both the nature and classification of variants, which sometimes does not follow evidence‐based standards (George et al. 2008; Thompson et al. 2014; Vail et al. 2015). CentoMD^®^ fulfills all of these requirements, although a number of variant databases exist that can also assist in variant evaluation and classification. These include: the HGMD^®^ (Stenson et al. 2014), a manually curated comprehensive source of information on germline disease‐causing variants and disease‐associated polymorphisms based on peer‐reviewed literature; the OMIM database (Amberger et al. 2015), which is similar in scope to HGMD^®^ but includes significantly fewer variants and focuses on phenotype descriptions; ClinVar (Landrum et al. 2014), an open‐access public archive of reports that lists relationships between human variations and phenotypes with supporting evidence; the Catalogue of Somatic Mutations in Cancer (COSMIC) (Forbes et al. 2011); and an assorted collection of locus‐specific databases (http://www.hgvs.org/dblist/glsdb.html).

Conceptually, the two most similar alternatives to CentoMD^®^ are ClinVar, which is freely available, and HGMD^®^, which is a pay‐per‐use resource. Although these two options provide genome‐wide, comprehensive collections of annotated and classified sequence variants linked to human phenotypes, they are also affected by well‐known limitations in their concept and design. For instance, ClinVar aggregates data from different sources, including unpublished (nonpeer‐reviewed) information, but it does not verify assertions nor endorse the accuracy or quality of variant classifications. Multiple different assertions can be given for the same variant from independent submitters, and very often these are not accompanied by sufficient evidence to support any interpretation of clinical significance as this is still an optional field for ClinVar submission. As a consequence, the quality of assertions and supporting evidence in ClinVar are dependent on the submitter and vary among entries (Johnston and Biesecker 2013). This can lead to the inconsistent determination of pathogenicity for the same variant across submitters (Rehm et al. 2015).

The HGMD^®^ is primarily based on manually curated, peer‐reviewed literature review published mainly in Europe and the US, and therefore focusing on the Caucasian population. The HGMD^®^ therefore relies mostly on external judgments from authors, reviewers, and editors of the source publications. It does not contain either unpublished data or somatic or mitochondrial variants. Further, no zygosity information is provided for individual variants. The HGMD^®^ also has two other major restrictions: (1) the quality of publications are not questioned and are sometimes misinterpreted, leading to incorrect variant classification; and (2) transfer of variants to the database has a restricted quality control, leading in some instances to opposite classifications (e.g., variants described as neutral in a publication are reported as pathogenic in the HGMD^®^). Finally, the public version is only updated twice per annum and is permanently 3 years out‐of‐date. Thus, the public version of the HGMD^®^ is not sufficiently up‐to‐date for clinical diagnostics in most cases, which makes it necessary to subscribe to HGMD^®^ Professional to access up‐to‐date information (Johnston and Biesecker 2013; Stenson et al. 2014; Vail et al. 2015).

As an alternative or addition to the databases discussed above, CentoMD^®^ v3.1 (released September 2016) covers more than 2500 disease genes including nuclear coding, nuclear noncoding, and mitochondrial genes. The data in this database are fully anonymized and the link between individuals and variants is thus removed. For all genetic variants, the demographic and phenotype information of the associated patients and probands are displayed. This includes year of sample analysis, age at diagnosis, gender, country of sample origin, pedigree, and clinical information. All patient phenotypes have been mapped to vocabulary defined by HPO criteria (Köhler et al. 2014), which allows the accurate and detailed collection and exchange of phenotype information that is critical for assessing human variation (Rehm et al. 2015). The HPO system provides users with a more accurate and powerful semantic phenotype search. This offers a comprehensive summary of the associated clinical picture and supporting evidence for the annotation class determined for each variant. Importantly, the patient cohort forming the database is representative of the global population. CentoMD^®^ curators continuously reassess the curated database content to keep variant data up‐to‐date. Thus, in addition to newly added variants, old variant entries may be revised or even recategorized based on additional, important new information in every quarterly release cycle.

The ultimate goal of genetic testing is to identify the etiology of a suspected genetic disorder for use in counseling and provision of tailored medical care (Vail et al. 2015). Gene variant classification must therefore be accurate and consistent in accordance with international, professional guidelines. The CentoMD^®^ system provides information about genotype–phenotype correlations based on tested clinical cases, with quality‐controlled variant annotations derived from stringent curation by medical professionals. This database contains not only genetic variant data that have already been reported to cause a particular disease or condition, but also a significant amount (56.9%) of relevant, newly detected variants that have never been described or reported as being associated with, or causing, any abnormal phenotypes.

Perhaps most importantly, the database constantly reclassifies variants based on updated phenotype information, family anamneses, and allele frequencies observed in the population analyzed by Centogene AG and in public databases. In version 3.1, 2.7% of variants that have previously been published as likely disease‐causing variants have in fact been proven to be likely benign based on available clinical evidence. In addition, users receive notifications of changes to the classified clinical significance with every release, thereby reducing the risk of data misinterpretation and providing the most up‐to‐date types of supporting evidence.

As with all genotype–phenotype databases, the CentoMD^®^ database faces a number of challenges that will need to be met as it develops over the coming years. In particular, data entry and curation will need to keep up with expected growth due to completion of WGS analyses. Automation of the curation process will need to be developed, and alignment with external data sources, and maintenance of the accuracy of phenotype description (and links with genotypes) will also need to be addressed.

In conclusion, this new database, which is accessible to accredited healthcare professionals according to a pay‐per‐use model, combines patient information, detailed clinical descriptions, genetic variants, and observed enzymatic activities and biomarker information (when available), and offers a comprehensive summary of the clinical validity and causality of detected gene variants with their associated phenotypes. CentoMD^®^ is a constantly evolving and growing accessible knowledge base with the chief aim of becoming the most comprehensive genotype–phenotype information resource for medically relevant gene variants.

## Conflict of Interest

DT, GEO, YS, and ABA are employees of Centogene AG; RAJ has been a former employee of the company; AR is founder and CEO of the company and holds shares of Centogene AG.

## References

[mgg3262-bib-0001] Abecasis, G. R. , A. Auton , L. D. Brooks , M. A. DePristo , R. M. Durbin , R. E. Handsaker , et al. 2012 An integrated map of genetic variation from 1,092 human genomes. Nature 491:56–65. 23128226 10.1038/nature11632 PMC3498066

[mgg3262-bib-0002] Adams, D. R. , M. Sincan , K. Fuentes Fajardo , J. C. Mullikin , T. M. Pierson , C. Toro , et al. 2012 Analysis of DNA sequence variants detected by high‐throughput sequencing. Hum. Mutat. 33:599–608. 22290882 10.1002/humu.22035 PMC3959770

[mgg3262-bib-0003] Amberger, J. S. , C. A. Bocchini , F. Schiettecatte , A. F. Scott , and A. Hamosh . 2015 OMIM.org: Online Mendelian Inheritance in Man (OMIM^®^), an online catalog of human genes and genetic disorders. Nucleic Acids Res. 43(Database issue):D789–D798. 25428349 10.1093/nar/gku1205 PMC4383985

[mgg3262-bib-0004] Baglioni, C. 1962 The fusion of two peptide chains in hemoglobin Lepore and its interpretation as a genetic deletion. Proc. Natl Acad. Sci. USA 48:1880–1886. 13968954 10.1073/pnas.48.11.1880 PMC221090

[mgg3262-bib-0005] Conter, C. , M. O. Rolland , D. Cheillan , V. Bonnet , I. Maire , and R. Froissart . 2006 Genetic heterogeneity of the GLDC gene in 28 unrelated patients with glycine encephalopathy. J. Inherit. Metab. Dis. 29:135–142. 16601880 10.1007/s10545-006-0202-6

[mgg3262-bib-0006] Cotton, R. G. , K. Phillips , and O. Horaitis . 2007 A survey of locus‐specific database curation. Human Genome Variation Society. J. Med. Genet. 44:e72. 17400791 10.1136/jmg.2006.044081 PMC2598041

[mgg3262-bib-0007] Deng, Y. , L. Gao , B. Wang , and X. Guo . 2015 HPOSim: an R package for phenotypic similarity measure and enrichment analysis based on the human phenotype ontology. PLoS ONE 10:e0115692. 25664462 10.1371/journal.pone.0115692 PMC4321842

[mgg3262-bib-0008] den Dunnen, J. T. , and S. E. Antonarakis . 2000 Mutation nomenclature extensions and suggestions to describe complex mutations: a discussion. Hum. Mutat. 15:7–12. 10612815 10.1002/(SICI)1098-1004(200001)15:1<7::AID-HUMU4>3.0.CO;2-N

[mgg3262-bib-0009] Forbes, S. A. , N. Bindal , S. Bamford , C. Cole , C. Y. Kok , D. Beare , et al. 2011 COSMIC: mining complete cancer genomes in the Catalogue of Somatic Mutations in Cancer. Nucleic Acids Res. 39(Database issue):D945–D950. 20952405 10.1093/nar/gkq929 PMC3013785

[mgg3262-bib-0010] George, R. A. , T. D. Smith , S. Callaghan , L. Hardman , C. Pierides , O. Horaitis , et al. 2008 General mutation databases: analysis and review. J. Med. Genet. 45:65–70. 17893115 10.1136/jmg.2007.052639

[mgg3262-bib-0011] Hardison, R. C. , D. H. Chui , C. R. Riemer , W. Miller , M. F. Carver , T. P. Molchanova , et al. 1998 Access to a syllabus of human hemoglobin variants (1996) via the World Wide Web. Hemoglobin 22:113–127. 9576329 10.3109/03630269809092136

[mgg3262-bib-0012] Hennekam, R. C. , and L. G. Biesecker . 2012 Next‐generation sequencing demands next‐generation phenotyping. Hum. Mutat. 33:884–886. 22457028 10.1002/humu.22048 PMC3327792

[mgg3262-bib-0013] Johnston, J. J. , and L. G. Biesecker . 2013 Databases of genomic variation and phenotypes: existing resources and future needs. Hum. Mol. Genet. 22(R1):R27–R31. 23962721 10.1093/hmg/ddt384 PMC3782073

[mgg3262-bib-0014] Köhler, S. , S. C. Doelken , C. J. Mungall , S. Bauer , H. V. Firth , I. Bailleul‐Forestier , et al. 2014 The Human Phenotype Ontology project: linking molecular biology and disease through phenotype data. Nucleic Acids Res. 42(Database issue):D966–D974. 24217912 10.1093/nar/gkt1026 PMC3965098

[mgg3262-bib-0015] Kure, S. , K. Kato , A. Dinopoulos , C. Gail , T. J. DeGrauw , J. Christodoulou , et al. 2006 Comprehensive mutation analysis of GLDC, AMT, and GCSH in nonketotic hyperglycinemia. Hum. Mutat. 27:343–352. 16450403 10.1002/humu.20293

[mgg3262-bib-0016] Landrum, M. J. , J. M. Lee , G. R. Riley , W. Jang , W. S. Rubinstein , D. M. Church , et al. 2014 ClinVar: public archive of relationships among sequence variation and human phenotype. Nucleic Acids Res. 42(Database issue):D980–D985. 24234437 10.1093/nar/gkt1113 PMC3965032

[mgg3262-bib-0017] Rehm, H. L. , J. S. Berg , L. D. Brooks , C. D. Bustamante , J. P. Evans , M. J. Landrum , et al. 2015 ClinGen–the Clinical Genome Resource. N. Engl. J. Med. 372:2235–2242. 26014595 10.1056/NEJMsr1406261 PMC4474187

[mgg3262-bib-0018] Richards, S. , N. Aziz , S. Bale , D. Bick , S. Das , J. Gastier‐Foster , et al. 2015 Standards and guidelines for the interpretation of sequence variants: a joint consensus recommendation of the American College of Medical Genetics and Genomics and the Association for Molecular Pathology. Genet. Med. 17:405–424. 25741868 10.1038/gim.2015.30 PMC4544753

[mgg3262-bib-0019] Rolfs, A. , F. Fazekas , U. Grittner , M. Dichgans , P. Martus , M. Holzhausen , et al. 2013 Acute cerebrovascular disease in the young: the Stroke in Young Fabry Patients study. Stroke 44:340–349. 23306324 10.1161/STROKEAHA.112.663708

[mgg3262-bib-0020] Stenson, P. D. , M. Mort , E. V. Ball , K. Shaw , A. Phillips , and D. N. Cooper . 2014 The Human Gene Mutation Database: building a comprehensive mutation repository for clinical and molecular genetics, diagnostic testing and personalized genomic medicine. Hum. Genet. 133:1–9. 24077912 10.1007/s00439-013-1358-4 PMC3898141

[mgg3262-bib-0021] Thompson, B. A. , A. B. Spurdle , J. P. Plazzer , M. S. Greenblatt , K. Akagi , F. Al‐Mulla , et al. 2014 Application of a 5‐tiered scheme for standardized classification of 2,360 unique mismatch repair gene variants in the InSiGHT locus‐specific database. Nat. Genet. 46:107–115. 24362816 10.1038/ng.2854 PMC4294709

[mgg3262-bib-0022] Vail, P. J. , B. Morris , A. van Kan , B. C. Burdett , K. Moyes , A. Theisen , et al. 2015 Comparison of locus‐specific databases for BRCA1 and BRCA2 variants reveals disparity in variant classification within and among databases. J. Community Genet. 6:351–359. 25782689 10.1007/s12687-015-0220-x PMC4567983

